# The Interplay Between Prenatal Adversity, Offspring Dopaminergic Genes, and Early Parenting on Toddler Attentional Function

**DOI:** 10.3389/fnbeh.2021.701971

**Published:** 2021-07-29

**Authors:** Eszter Szekely, Alexia Jolicoeur-Martineau, Leslie Atkinson, Robert D. Levitan, Meir Steiner, John E. Lydon, Alison S. Fleming, James L. Kennedy, Ashley Wazana

**Affiliations:** ^1^Department of Psychiatry, McGill University Faculty of Medicine, Montreal, QC, Canada; ^2^Lady Davis Institute for Medical Research, Jewish General Hospital, Montreal, QC, Canada; ^3^MILA—Quebec Artificial Intelligence Institute, Montreal, QC, Canada; ^4^Department of Computer Sciences, Université de Montréal, Montreal, QC, Canada; ^5^Department of Psychology, Ryerson University, Toronto, ON, Canada; ^6^Centre for Addiction and Mental Health, Toronto, ON, Canada; ^7^Department of Psychiatry, University of Toronto, Toronto, ON, Canada; ^8^Department of Psychiatry and Behavioural Neurosciences, McMaster University, Hamilton, ON, Canada; ^9^Department of Psychology, McGill University, Montreal, QC, Canada; ^10^Department of Psychology, University of Toronto Mississauga, Toronto, ON, Canada; ^11^Centre for Child Development and Mental Health, Jewish General Hospital, Montreal, QC, Canada

**Keywords:** attention, child, prenatal adversity, dopamine, genes, maternal sensitivity to infant cues, parenting (MeSH)

## Abstract

**Background**: Few studies have explored the complex gene-by-prenatal environment-by-early postnatal environment interactions that underlie the development of attentional competence. Here, we examined if variation in dopamine-related genes interacts with prenatal adversity to influence toddler attentional competence and whether this influence is buffered by early positive maternal behavior.

**Methods**: From the Maternal Adversity, Vulnerability and Neurodevelopment cohort, 134 participants (197 when imputing missing data) had information on prenatal adversity (prenatal stressful life events, prenatal maternal depressive symptoms, and birth weight), five dopamine-related genes (*DAT1, DRD4, DRD2, COMT, BDNF*), observed maternal parenting behavior at 6 months and parent-rated toddler attentional competence at 18 and 24 months. The Latent Environmental and Genetic Interaction (LEGIT) approach was used to examine genes-by-prenatal environment-by-postnatal environment interactions while controlling for sociodemographic factors and postnatal depression.

**Results**: Our hypothesis of a three-way interaction between prenatal adversity, dopamine-related genes, and early maternal parenting behavior was not confirmed. However, consistent two-way interactions emerged between prenatal adversity and dopamine-related genes; prenatal adversity and maternal parenting behavior, and dopamine-related genes and maternal parenting behavior in relation to toddler attentional competence. Significant interaction effects were driven by the *DAT1, COMT*, and *BDNF* genotypes; prenatal stressful life events; maternal sensitivity, tactile stimulation, vocalization, and infant-related activities.

**Conclusions**: Multiple dopamine-related genes affected toddler attentional competence and they did so in interaction with prenatal adversity and the early rearing environment, separately. Effects were already visible in young children. Several aspects of early maternal parenting have been identified as potential targets for intervention.

## Introduction

There is increasing evidence that an adverse prenatal environment contributes to the risk of developing attention-deficit/hyperactivity disorder (ADHD; Banerjee et al., 2007; Thapar and Rutter, 2009). The most commonly studied prenatal risks of ADHD are maternal lifestyle factors, such as smoking, alcohol consumption, substance use, and severe stress/anxiety experienced during pregnancy (Fleming et al., 1988; Banerjee et al., 2007; Li et al., 2010). Further, low birth weight and prematurity at birth—as indicators of a suboptimal intrauterine environment—have also been implicated in the risk for ADHD, particularly inattention symptoms (Bhutta et al., 2002; Strang-Karlsson et al., 2008). However, much less is known about the role of prenatal maternal depression in the development of offspring ADHD symptoms. This is important given that approximately 40% of mothers of children with ADHD have a history of major depression, making them 2–3 times more likely to be depressed than women in the general population (Chronis-Tuscano et al., 2003; Kessler et al., 2006). Furthermore, prenatal depression is consistently linked to shorter gestation and lower birth weight, which are both common risk factors of ADHD (Field et al., 2006; Field, 2011). The available literature suggests that maternal depressive symptoms during pregnancy can negatively shape the offspring’s attention system and increase the risk of comorbidity in those children who already have a diagnosis of ADHD (Chronis-Tuscano et al., 2010; Van Batenburg Eddes et al., 2013). Based on the above, in the present study we capture prenatal adversity in three important ways: through the number of stressful life events experienced by women during pregnancy, maternal depressive symptoms during pregnancy, and birth weight of children.

The considerable variability in neurodevelopmental outcomes among children who experience prenatal adversity indicates potential differences in children’s vulnerability to the environment. Previous research has highlighted the importance of genetic factors in conferring such vulnerability (Caspi et al., 2002; Rutter, 2006; Laucht et al., 2007). Indeed, gene-environment interactions (G × E) are increasingly recognized as important contributors to the emergence of psychopathology (Caspi and Moffitt, 2006; Rutter, 2006; Belsky et al., 2009). Yet, there have been few published studies examining the contribution of G × E effects to ADHD (Thapar et al., 2007; Nigg, 2012) and even fewer that have specifically focused on the prenatal environment (for a review, see Franke and Buitelaar, 2018). Not surprisingly, these studies have mainly focused on dopaminergic genes, as both pharmacological and genetic research suggest a critical role for dopamine in attentional, motivational, and exploratory neurobehavioral processes (Faraone et al., 2005; Thapar et al., 2007). Regarding attention in particular, animal studies have suggested a direct link between selective lesions of dopaminergic neurons and altered attentional processes in rodents and primates (Nieoullon, 2002; Thiele and Bellgrove, 2018). Based on these studies, the specific attention components that were most affected included selective attention, spatial attention, detection of novelty, and sustained attention (for a review, see Nieoullon, 2002). Notably, the exact result of lesioning dopaminergic neurons in different brain regions depended on the nature of the brain area concerned. As dopamine is mainly present in the frontal cortex and basal ganglia in the brain, it is hypothesized that attention deficits might confer alterations in these subcortical brain structures closely linked to cortical regions rather than simple alterations in dopaminergic transmission (Nieoullon, 2002). Thus, behavioral changes following cortical dopamine depletion have to be interpreted in light of any associated changes in dopaminergic transmission at a subcortical level (Nieoullon, 2002). For instance, methylphenidate, a drug that is most commonly used in the treatment of ADHD by modulating dopaminergic transmission, was found to equally increase frontal cortical activity in both healthy controls and children with ADHD during a response inhibition task, whereas it increased striatal activity only in children with ADHD and decreased it in healthy controls (Vaidya et al., 1998). More directly relevant to our study, genetic variation linked to dopaminergic transmission in both the frontal cortex and related subcortical regions impacted infant attention at age 9 months (Holmboe et al., 2010).

### Dopaminergic Genes by Prenatal Adversity Interaction Effects on ADHD

The dopamine transporter *DAT1* gene has been a prime candidate for research in this context. The gene codes for a solute carrier protein responsible for the reuptake of dopamine from the synaptic cleft to the presynaptic neuron. This protein is densely present in the striatum and nucleus accumbens and constitutes the primary mechanism of dopamine regulation in these brain regions (Ciliax et al., 1999). The most widely studied *DAT1* polymorphism is a variable number tandem repeat (VNTR) sequence in the 3′ untranslated region that is 40 base pairs (bp) in length (Vandenbergh et al., 1992). The most common alleles are the 10 (480-bp; 71.9%) and 9 (440-bp; 23.4%) repeats (Doucette Stamm et al., 1995). This polymorphism is believed to be functional, influencing dopamine transporter availability and binding potential (Gizer et al., 2009) and is associated with sustained attention (Loo et al., 2003). *DAT1* has been found to interact with prenatal maternal smoking (Brookes et al., 2006; Neuman et al., 2007), alcohol consumption (Kahn et al., 2003), and family adversity (Laucht et al., 2007) to increase the risk for ADHD. Some studies reported that the DAT1-prenatal maternal smoking interactions were significant only in boys homozygous for the 10-repeat allele and only for hyperactive-impulsive symptoms (Altink et al., 2008; Becker et al., 2008), while another, smaller study found no interaction effect for *DAT1* and prenatal maternal smoking on ADHD (Langley et al., 2008).

Another popular candidate for G × E studies on ADHD is the dopamine receptor D4 gene (*DRD4*), specifically a 48bp VNTR on exon 3. *DRD4* is predominantly expressed in the frontal lobe, such as the orbitofrontal cortex and anterior cingulate (Floresco and Maric, 2007). The most common alleles of this polymorphism are the 2-, 4-, and 7-repeat alleles, although this varies significantly across ethnic groups (Chang et al., 1996). This VNTR is likely functional in that the 7-repeat allele slightly differs from the 2- and 4-repeat alleles in secondary messenger activity and in response to clozapine, an antipsychotic medication (Asghari et al., 1994, 1995). The VNTR has further been found to influence sustained attention and information processing from an early age (Auerbach et al., 2001; Fan et al., 2003). In terms of its interaction with the environment, results suggest that the 7-repeat allele of *DRD4* exacerbates the effects of prenatal adversity, as reflected in increased risk for ADHD and more severe ADHD symptoms (Grizenko et al., 2012). One study found similar relations but only in the case of teacher-reported inattention symptoms rather than parent-reported ADHD symptoms (Altink et al., 2008). Another, smaller study reported a lack of significant G × E between *DRD4* and any measures of prenatal adversity (i.e., maternal smoking, alcohol use, or child’s birth weight (Langley et al., 2008).

An additional dopaminergic gene that has been examined in relation to environmental adversity and ADHD is the dopamine receptor D2 (*DRD2*) gene (Ficks and Waldman, 2009). *DRD2* is expressed in the basal ganglia and prefrontal cortex and is key in regulating the mesolimbic reward system (Usiello et al., 2000). Studies of *DRD2* have tended to focus on a TaqIA restriction site (rs1800497), downstream from *DRD2* located in an exon of a neighboring gene, *ANKK1* (Neville et al., 2004; Grizenko et al., 2012). Nonetheless, this polymorphism is known to influence *DRD2* expression levels (Gluskin and Mickey, 2016). DRD2 has been implicated in affecting selective attention in patients with schizophrenia (Nkam et al., 2017). In terms of G × E interactions involving *DRD2*, ADHD was more prevalent among children whose mothers experienced less stable marital environments (i.e., having had no or multiple marriages) only if they were homozygous for the TaqI-A2 allele (Waldman, 2007).

Another important gene that has been studied in a G × E framework in ADHD is the catechol-O-methyltransferase (*COMT*), which is involved in the degradation catecholamines, such as dopamine. *COMT* has a particularly important role in the frontal cortex, where it accounts for approximately 50–60% of the metabolic degradation of dopamine (Karoum et al., 1994). The gene includes a common functional polymorphism with a methionine (“*met*”) to valine (“*val*”) substitution at codon 158. The *met* allele is associated with low enzyme activity, while the *val* allele is associated with high enzyme activity (Chen et al., 2004). This polymorphism has been implicated in relation to distractibility (Holmboe et al., 2010) and attentional control (Goldberg and Weinberger, 2004; Blasi et al., 2005; Ciampoli et al., 2017). Regarding its interaction with prenatal adversity among children with ADHD, one study found that those who carried the *COMT* val/val genotype (for rs4680) were more susceptible to the adverse effects of prenatal risks as indexed by lower birth weight to develop early-onset antisocial behavior (Thapar et al., 2005). Furthermore, in a combined analysis of two large cohorts (ALSPAC and PREDO) there was a robust interaction effect of child *COMT* (val/val rs4680) genotype with maternal prenatal anxiety to predict ADHD symptoms assessed at multiple time points (O’Donnell et al., 2017).

Finally, significant G × E effects have also been reported for inattention symptoms involving the brain-derived neurotrophic factor (*BDNF*) gene, which, besides being a regulator of neuronal development and function, plays a role in dopamine neurotransmission (Guillin et al., 2001; Narita et al., 2003). *BDNF* exerts influence on the brain’s mesolimbic and corticolimbic reward pathways by modulating their response to dopamine (Guillin et al., 2001). A common polymorphism on the *BDNF* gene in which a valine is replaced by a methionine at codon 66 (Val66Met; rs6265) has been shown to influence the intracellular trafficking and activity-dependent secretion of *BDNF* in brain (Chen et al., 2004). The BDNF gene has been associated with general cognitive performance (Dincheva et al., 2012). Within a G × E context, Lasky-Su et al. (2007) found that in lower SES environments children (6–18-year-old) carrying the risk alleles of rs1013442, rs1387144, or Val66Met was associated with having more inattention symptoms.

### Parent-Child Interactions and Their Influence on ADHD

Notably, and perhaps more importantly for clinicians, certain environmental factors have the potential to modify the impact of prenatal adversity in genetically susceptible children (Thomas et al., 2015). Parenting, for instance, is a robust environmental predictor of developmental outcomes in children with ADHD (Deault, 2010). While positive parenting can protect against developing comorbidity in children with ADHD—even when exposed to maternal depression (Chronis-Tuscano et al., 2007)—negative parenting has been associated with elevated ADHD symptomatology above and beyond shared genetic effects (Harold et al., 2013). High levels of negativity in parent-child interactions or reciprocal coercive communication are common in families of children with ADHD (Danforth et al., 1991; Pfiffner et al., 2005; Romirowsky and Chronis-Tuscano, 2014). Sensitive parenting may be particularly effective at buffering the negative effects of prenatal adversity on child cognition and behavior (Laucht et al., 2001; Plamondon et al., 2015; Pickles et al., 2017). Randomized clinical control trials found that parent training promoting positive parent-child interactions was effective in ADHD (Young and Amarasinghe, 2010). Although the exact mechanisms are currently unknown, there is an indication that the positive effects of a more sensitive/less intrusive parenting style on ADHD may be indirect, by supporting the development of protective mechanisms, such as inhibitory control mechanisms in children (Miller et al., 2019). Importantly, the general recommendation is that for preschool children showing signs of ADHD parent training should be the first line of treatment, and medication introduced only in case when parent training is not effective (Daley et al., 2009; Pelham et al., 2016). Thus, the literature suggests that parents have a key role in the development of their children’s attention skills (Gauvain, 2001; Davis and Williams, 2011). Attentional competencies develop through dynamic and continuous interactions between the child and their physical and social surroundings (Vygotsky, 1978; Landry et al., 2002). In this process, parents initially regulate their child’s attention through supportive parenting or “scaffolding” until children are able to regulate their own attentional processes (Conner et al., 1997; Gauvain et al., 2001). Failure to develop appropriate attention regulation skills in early childhood can have lasting effects on later development and academic success (Blair, 2002). Although during the preschool years it may be challenging to differentiate delayed regulatory skills from true ADHD, research suggests that, in both cases, parent-child interactions may be key to minimizing later adverse outcomes (Davis and Williams, 2011).

### Statistical Issues in Modeling Gene-by-Environment Interaction Effects

To date, most G × E studies on ADHD (and other psychiatric disorders) have considered a single genetic variant and a single environmental exposure at a time, which significantly limits the explanatory value of G × E models for complex phenotypes, such as ADHD. These G × E models often have very small effect sizes and low replication rates (Risch et al., 2009; Lee et al., 2012). One recommended strategy to overcome this limitation is to simultaneously examine multiple candidate genes affecting the same biological pathway (e.g., dopaminergic transmission) as well as multiple relevant environmental factors (Pennington and Bishop, 2009). In a review, Pennington and Bishop (2009) suggested computing composite G and E risk scores across candidate genes and environmental factors and test for G and E main effects and G × E interactions in one omnibus analysis. Then, in case of a significant interaction effect, follow-up analyses should be performed to specify which risk alleles and which environments contribute to the overall effect. However, until now there has been a lack of appropriate statistical methodology to perform such multi-G × E analyses. We recently developed a method for the analysis of complex interactions between multiple genes and environments (Jolicoeur-Martineau et al., 2019, 2020). The Latent Environmental and Genetic InTeraction (LEGIT) approach can be used to construct complex multi-interaction models without the need to estimate an additional parameter for each interaction term, thus improving scalability, especially with higher order interactions. An important limitation of previous G × E models is the lack of information concerning the specific form of the interaction effect (Widaman et al., 2012). For instance, the *diathesis-stress* model assumes that the differences between individuals with and without the “risk” allele of a given genetic variant will manifest only under adverse circumstances, such that individuals carrying the “risk” allele are affected negatively, while those without the “risk” allele remain relatively unaffected by the environment (Belsky et al., 2009). In comparison, the *differential-susceptibility* model posits that individuals carrying the “risk” allele are generally more sensitive to the effects of the environment than those without the “risk” allele (Belsky, 1997; Boyce and Ellis, 2005). Accordingly, compared to those with the non-risk allele, individuals with the “risk” allele exhibit poorer outcomes in negative environments, similar outcomes in average environments, and superior outcomes in positive environments. The LEGIT approach allows us to distinguish between these two theoretical frameworks, which may have important consequences for prevention and intervention strategies.

Here, we use a rich longitudinal dataset to examine how dopaminergic candidate genes simultaneously interact with prenatal adversity, and early parenting to influence toddlers’ attentional competence measured longitudinally at two time points. We apply LEGIT—with a G × E_1_ × E_2_ design to address this question. Our findings may advance the literature in three important ways. First, we examine the effect of prenatal adversity by including a number of well-established measures of prenatal adversity in one model. Second, we simultaneously consider the modifying effect of multiple dopamine-related genes known to affect the developing human attention system. Third, we complement this by additionally examining important aspects of the early rearing environment that may buffer the negative effects of prenatal adversity in genetically susceptible children. We address these questions using an approach that was specifically designed to deal with the complexity of simultaneously testing multiple interaction effects in relation to an outcome. Due to methodological limitations, few studies to date have attempted to look at the joint contribution of multiple genetic risk variants and multiple environmental exposures (both adverse and protective) to early attention development. This, however, seriously limits our understanding of complex human behavior, which is underlined by the interplay of numerous biological and environmental factors. One novelty of this study is thus the use of LEGIT that enabled the simultaneous testing of a large number of G × E interactions by using latent genetic and environmental features and an alternating optimization algorithm. Another novelty of our study is the inclusion of both macro- and micro-level analytic observations of early maternal behavior. Maternal behaviors included here were analyzed on a second-by-second level within the context of a 20-min mother-infant interaction. Given the time- and labor-intensive nature of collecting such fine-grained data, we are not aware of many G × E studies on early child attention that have used observational measures of early parenting, furthermore both at a macro- and micro-analytic level.

## Materials and Methods

### Participants

The participants were mother-child dyads from the Maternal Adversity, Vulnerability and Neurodevelopment (MAVAN) project, a Canadian community-based prenatal cohort of 590 women and their children in Montreal (Quebec) and Hamilton (Ontario). Women were recruited in maternity hospitals from 2003 to 2009 during their routine ultrasound examinations. A detailed description of the cohort has been presented elsewhere (O’Donnell et al., 2014). Informed consent was obtained at the time of recruitment and at each time point of data acquisition. Ethics Review Board approval was obtained from the institution of each study site. Retention rates for the MAVAN subjects were 97.4% at 6 months, 84.0% at 18 months, and 80.6% at 36 months. The present study included 134 mother-child dyads with complete data either at 18 or 24 months of child age. The reduction of sample size from 577 to 134 participants is explained by the following: 240 participants had missing genotype data (due to partial funding), 61 participants had missing information on prenatal adversity; seven participants had missing data regarding early parenting; 86 participants had missing information on postnatal maternal depression; 49 participants had missing outcome data at both 18 and 24 months. Thus, the final sample for the complete case analysis included 134 women and their children.

### Measures

#### Genotyping

Child genotype was obtained from buccal swabs. using the TaqMan methods on the ABI-7000 for single nucleotide polymorphism (SNP) markers and ABI-3100 for repeat polymorphisms. To ensure a clear result, any ambiguous genotypes were discarded and the subjects were re-genotyped until the results were unambiguous. Each 20th marker was re-genotyped to check for error rates (0.5%). For the present study, we were interested specifically in genes directly or indirectly related to the dopaminergic system. The five candidate genes included dopamine receptors *DRD2, DRD4*, dopamine transporter *DAT1*, the catechol-o-methyltransferase (*COMT*), and the brain-derived neurotrophic factor (*BDNF*). *DRD2* was captured using SNP rs1800497 (also known as TaqIA) with A as the risk allele (Nyman et al., 2007; Moro et al., 2019). *COMT* was captured using SNP rs4680 with Met as the risk allele (Holmboe et al., 2010; Soeiro-De-Souza et al., 2013). *BDNF* was captured using SNP rs6265 with Val as the risk allele. These SNPs were coded as the number of “risk” alleles divided by two (i.e., 0 for no risk allele, 0.5 for one risk allele, 1 for two risk alleles). *DAT1* was captured using the 40bp VN TR located in exon 15 coded dichotomously as 1 (10R/10R) and 0 when 9R/9R or 9R/10R (Cornish et al., 2005; Holmboe et al., 2010). *DRD4* was captured using the 48bp (VN TR) polymorphism in exon 3 coded dichotomously as 1 (6-8R) and 0 (2-5R), as per Schmidt et al. (2001). Genotype distributions did not deviate from Hardy-Weinberg equilibrium (*p* > 0.05).

#### Prenatal Life Events

An adapted version of the Prenatal Life Events Scale (Lobel, 1997; Lobel et al., 2000) was used to assess the occurrence of 17 life events (e.g., being robbed, being involved in a serious accident, having someone close die) that women may have experienced during the pregnancy (24–36 weeks). This adapted version did not include those items from the original version of the scale that had an especially low frequency of occurrence. For each event endorsed, participants reported how undesirable or negative the event was on a scale from 0 (not at all) to 3 (very much). Life events that were evaluated as strongly undesirable (i.e., score of 2 or 3) were coded as 1, everything else was coded as 0. Scores were summed to quantify the number of stressful life events. Total scores ranged from 0 to 17, with higher scores indicating the presence of more stressful life events during pregnancy. Not surprisingly, the internal reliability of this scale was low (*α* = 0.42) due to the wide range of possibly unrelated life events.

#### Prenatal Depressive Symptoms

Women rated their depressive symptoms at 24–36 weeks of pregnancy using the Center for Epidemiologic Studies Depression Scale (CES-D; Radloff, 1977) The CES-D includes 20 items capturing mood-, appetite- and sleep-related symptoms in community-based populations. Each item was rated on a scale from 0 (rarely or none of the time) to 3 (most or all of the time) and the items were summed. Total scores ranged from 0 to 60, with higher scores indicating more severe depressive symptoms. Internal reliability of the CES-D in the present sample was high (*α* = 0.92).

#### Birth Weight

Children’s weight at birth was assessed at the time of delivery (in grams).

#### Maternal Sensitivity and Parenting Behaviors

When children were 6 months old, maternal sensitivity and maternal parenting behaviors were observed during a 20-min free-play session, which took place in the participant’s home and was videotaped for coding purposes. We assessed maternal sensitivity using the Ainsworth Maternal Sensitivity Scales (Ainsworth et al., 1978). This is a validated gold standard, macro-analytic-level measure of maternal sensitivity, focusing on four aspects of early care: sensitivity to infant signals, cooperation vs. interference with ongoing behavior, psychological and physical availability, and acceptance vs. rejection of infant’s needs. Scores ranged from 1 to 9, with higher scores indicating more highly involved mothers. Mean inter-rater reliability (intra-class correlation) for the Ainsworth scale ratings was 0.88 (*n* = 28). The four scales were very highly correlated (*r* > 0.94). As such, we used only the sensitivity scale. Maternal parenting behaviors were assessed using the Behavioral Evaluation Strategies and Taxonomies (BEST; Educational Consulting, Inc. Florida, USA; S and K NorPark Computer Design, Toronto). The BEST consists of second-by-second micro-analytic-level frequency ratings and duration measures of maternal and child behaviors (Fleming et al., 1988). Two trained raters scored the duration and frequency of specific behaviors. Inter-rater reliabilities (intra-class correlation) ranged 0.74–0.90 (*n* = 18). Maternal sensitivity and BEST behaviors were coded independently, with coders of one scheme blind to codes on the other. Parental behaviors included maternal attention towards the child, tactile contact between mother and child, maternal vocalization, and mother-child activities. These measures have been used in our past research (Krpan et al., 2005; Giardino et al., 2008; Wazana et al., 2015; Graffi et al., 2018). For the purpose of this study, the duration of the respective maternal behaviors was first transformed into percentages of the total duration the mother spent interacting with her child, which excluded the time spent feeding, talking to someone else, or where the dyad was obscured. Percentages were subsequently z-standardized and averaged to form a score on the following dimensions:

1. Attention: focused (i.e., concentrated) looking at the infant, unfocused (i.e., unconcentrated) looking at infant or focused looking at an infant-related object (i.e., joint attention), “mother and infant are focusing on the same object”
2. Tactile stimulation: kissing, poking/tickling, mouthing/raspberries, stroking/patting
3. Vocalization: humming/singing, talking, laughing/smiling
4. Infant-related activities: social games, showing toy, play with a toy, play without a toy, rocking/jiggling, grooming the infant

#### Child Attentional Competency

Attentional competency was assessed using the Attention subscale from the Competence domain of the Infant-Toddler Social and Emotional Assessment (ITSEA) at 18 and 24 months (Briggs-Gowan and Carter, 1998, 2007). The ITSEA is a developmentally and clinically sensitive parent-rated questionnaire of social-emotional problems and competencies in 1–3 year-olds (Briggs-Gowan and Carter, 1998). The Attention subscale is formed by summing five items assessing attentional function, such as “plays with toys for 5 min or more,” “looks at things for a minute or longer.” Internal consistency of the Attention subscale in the present sample was good (Cronbach’s alpha = 0.76 at 18 months and 0.74 at 24 months). Scores were distributed evenly across the range of possible values (0–2) at both time points, values were higher at 24 months (*M* = 1.43, SD = 0.45) than 18 months (*M* = 1.29, SD = 0.51), with moderate consistency over time (*ICC*_(3,1)_ = 0.57, *ICC*_(3,k)_ = 0.73). Outcome scores were divided by 2 to rescale them between 0 and 1. Using a linear model (LM) with a constrained outcome variable is problematic as model predictions could go beyond the observed range. Therefore, we used a generalized linear model (GLM) with a Quasi-binomial family, which ensures that the outcome is constrained to the range [0, 1] instead of being unconstrained, such as when using a Gaussian family.

#### Covariates

Covariates included child sex, maternal age at delivery, and maternal education (“high school or less,” “some college, completed college, or some university,” and “university graduate or more”). We additionally included a covariate that indicated whether the child had available data on attentional competency at 24 months to adjust for the fact that baseline attentional competency was significantly better at 24 months (*β* = 0.18, *S* = 9,862.5, *p* < 0.0001). The intercept *β*_0_ of the model represents attentional competency at 18 months, while *β*_0_+ *β*_24M_ together represents attention competency at 24 months. All continuous variables were standardized except for maternal age.

### Statistical Analysis

#### Descriptives

Hardy-Weinberg equilibrium of genotype distribution was tested using exact tests (Engels, 2009). Since most continuous variables used in this study are non-normally distributed, we used non-parametric tests to describe the characteristics of our sample. We used chi-square tests for categorical-by-categorical, Wilcoxon rank-sum tests for binary-by-continuous, and Wilcoxon signed-rank tests for paired comparisons. We examined correlations between variables used in analyses using Kendall’s tau coefficients.

#### Main Analyses

Data were analyzed using the LEGIT R (Jolicoeur-Martineau et al., 2019) with a repeated measures design to predict attentional competency at 18 and 24 months. We fitted a 3-way G × E_1_ × E_2_ interaction model where G is a weighted sum (i.e., latent score) of the five dopamine-related candidate genes (i.e., *DRD2, DRD4, BNDF, COMT, DAT1*), E_1_ is a weighted sum of our three prenatal adversity variables (i.e., prenatal maternal stressful life events, prenatal maternal depressive symptoms, and birth weight), and E_2_ is a weighted sum of all early maternal parenting behaviors (i.e., Ainsworth sensitivity, maternal attention, tactile stimulation, vocalization, and infant-related activities). A schematic representation of the proposed three-way interaction model is shown in the Supplementary Figure 1. Further information on how the latent sum of G, E_1_, E_2_, and their interactions were calculated is provided as Supplemental Material.

#### Treatment of Missing Data

Missing information was imputed for participants that had at least one measure available for each latent score (i.e., G, E_1_, and E_2_), and the outcome variable at either 18 or 24 months (*N* = 197). All analyses were performed on both the complete cases (*N* = 134) and the imputed dataset (*N* = 197). Given that our model included interaction terms, traditional imputation methods which do not account for non-linearities are bound to be biased (Seaman et al., 2012). Thus, we used missForest (Stekhoven and Buhlmann, 2012), which has been shown to outperform the popular multiple imputation method by chained equations (mice) with predictive mean matching (pmm; van Buuren and Groothuis-Oudshoorn, 2010). Furthermore, the imputation accuracy of MissForest has been shown to approach state-of-the-art modern imputation techniques (Yoon et al., 2018; Payrovnaziri et al., 2020).

Similar to many complex, non-linear methods, it is not possible to pool estimates from multiple imputations using LEGIT. As the signs of the parameters inside the latent scores may differ randomly (models with the same parameters, but with different signs can be equivalent), pooling across multiple LEGIT models would lead to a regression of the parameters towards zero. Moreover, given the various parameters involved, it is difficult to know which sign is the correct one. All these can make pooling highly inconsistent, if not impossible. In addition, performing variable selection is unfeasible using multiple imputations. For the above reasons, we used a single imputation method called missForest. Contrary to other methods, such as mice, MissForest produces similar imputations when using different random seeds.

#### Variable Selection

To be more parsimonious, we can apply variable selection to retain only the most important elements in each latent score (G, E_1_, E_2_). Unfortunately, quality-of-fit measures like the Akaike information criterion (AIC; Akaike, 1998), corrected Akaike information criterion (AICc; Hurvich and Tsai, 1989), and Bayesian information criterion (BIC; Schwarz, 1978) are not defined in GLMs of quasi-binomial family. This means that we cannot use variable selection with these fit measures. Consequently, the variable selection was performed in the LM models, and the retained variables were included in the GLM models of the quasi-binomial family. Variables selected in the LM models generally remained significant in the GLM models, and their relative contribution did not change meaningfully. Variable selection was performed using “parallel natural evolutionary variable selection” available within LEGIT. Models with the lowest AICc value were considered as best fitting the data. Results from the models both with and without variable selection are presented.

#### In-Sample and Out-of-Sample Effect Sizes

To further assess model fit, we also examined the in-sample effect size and out-of-sample effect size. In-sample effect size was estimated using the regular *R*^2^, out-of-sample effect size (which measures how well the model generalizes to new observations) was estimated using the leave-one-out cross-validated (LOOCV) *R*^2^. The LOOCV was calculated in the same way as the *R*^2^ with the exception that the predictions for a given participant were obtained from a model that did not include the participant in question.

Data analysis was carried out in version 9.4 of the SAS System for Windows (Copyright © 2002–2012, SAS Institute Inc. SAS and all other SAS Institute Inc. product or service names are registered trademarks or trademarks of SAS Institute Inc., Cary, NC, USA). Graphical outputs and imputations were generated using R version 3.2.5 (R Development Core Team., 2016).

## Results

### Descriptive Analyses

Sample characteristics are shown in Table 1; correlations between the predictors, outcomes and covariates are shown in Table 2. Attentional competence at 18 and 24 months were highly correlated (*r*_(103)_ = 0.50, *p* < 0.0001). Attentional competence at 18 months was positively associated with maternal sensitivity (*r*_(120)_ = 0.14, *p* = 0.04) and negatively associated with postnatal depressive symptoms (*r*_(120)_ = −0.13, *p* = 0.04). Attentional competence at 24 months was negatively associated with birth weight (*r*_(117)_ = −0.14, *p* < 0.05), prenatal depressive symptoms (*r*_(117)_ = −0.22, *p* = 0.01), and postnatal depressive symptoms (*r*_(117)_ = −0.28, *p* = 0.0002). Prenatal depressive symptoms were positively associated with prenatal life events (*r*_(134)_ = 0.24, *p* < 0.0001) and postnatal depressive symptoms (*r*_(134)_ = 0.41 *p* < 0.0001). Maternal parenting measures were not significantly associated with each other, except for vocalization, which was positively related to infant-related activities (*r*_(134)_ = 0.16, *p* = 0.006) and maternal sensitivity (*r*_(134)_ = 0.19, *p* = 0.003). Significant gene-environment correlations were observed between *DRD2* and birth weight (*r*_(134)_ = −0.16, *p* = 0.02) and between *DRD4* and prenatal depressive symptoms (*r*_(134)_ = −0.15, *p* = 0.04) and vocalization (*r*_(134)_ = 0.15, *p* = 0.03).

**Table 1:** Demographic characteristics of MAVAN participants.

*N =* 134 (*n* = 103 at both time points, *n* = 17 at 18 months only, *n* = 14 at 24 months only)	M (SD) or N (%)
Maternal characteristics	
Age	30.46 (4.75)
Education	
High school or less and partial college	21 (15.67%)
Completed college or some university	
University graduate or higher	45 (33.58%)
Income (CAD)	
<15,000	68 (50.75%)
15,000–<30,000	
30,000–<50,000	4 (3.15%)
50,000–<80,000	16 (12.60%)
>80,000	30 (23.62%)
Prenatal depressive symptoms	36 (28.35%)
Postnatal depressive symptoms	41 (32.28%)
Maternal infant-related attention	11.94 (9.43)
Maternal tactile stimulation	11.06 (8.84)
Maternal vocalization	0.04 (0.29)
Maternal infant-related activities	−0.01 (0.51)
Maternal sensitivity	0.05 (0.47)
	0.03 (0.42)
	5.64 (1.92)
Child characteristics	
Gender (boys)	60 (44.78%)
Birth weight (g)	3329.02 (442.37)
DRD2 (Number of A1 alleles)	0: 85(63.43%), 1: 43(32.09%), 2: 6(4.48%)
DRD4	2–5 Repeat: 90 (67.16%), 6–8 Repeat: 44(32.84%)
DAT1	9R/9R or 9R/10R: 59(44.03%), 10R/10R: 75(55.97%)
BDNF (Number of Val Allele)	0: 90(67.16%) / 1: 38(28.36%) / 2: 6(4.48%)
COMT (Number of Met allele)	0: 35(26.12%) / 1: 71(52.99%) / 2: 28(20.90%)
Attentional competence at 18 months	1.29 (0.51)
Attentional competence at 24 months	1.43 (0.45)

**Table 2:** Kendall-Tau correlation matrix of all variables included in analyses.

Variables	1	2	3	4	5	6	7	8	9	10	11	12
1. Attention at 18 months	1	−	−	−	−	−	−	−	−	−	−	−
2. Attention at 24 months	0.50***	1	−	−	−	−	−	−	−	−	−	−
3. Prenatal depression	−0.05	−0.22***	1	−	−	−	−	−	−	−	−	−
4. Postnatal depression	−0.13*	−0.28***	0.41***	1	−	−	−	−	−	−	−	−
5. Prenatal life events	0.04	0.06	0.24***	0.14*	1	−	−	−	−	−	−	−
6. Birth weight (g)	−0.12	−0.14*	0.01	0.01	−0.06	1	−	−	−	−	−	−
7. Maternal age	0.08	0.09	−0.05	−0.06	−0.01	0.02	1	−	−	−	−	−
8. Maternal infant-related attention	0.05	−0.04	0.04	0.06	−0.03	−0.10	0.02	1	−	−	−	−
9. Maternal tactile stimulation	0.09	0.08	0.00	0.03	0.02	−0.08	−0.02	−0.02	1	−	−	−
10. Maternal Vocalization	0.05	0.10	−0.11	−0.01	0.01	−0.04	0.09	0.07	0.07	1	−	−
11. Maternal infant-related activities	0.08	−0.03	−0.02	0.02	−0.10	−0.04	−0.11	−0.04	0.06	0.16	1	−
12. Maternal sensitivity	0.14*	0.07	−0.07	−0.09	−0.06	0.09	0.09	0.08	0.07	0.19**	0.05	1
13. *DRD2* genotype	0.02	0.04	0.06	0.07	−0.07	−0.16*	0.11	0.11	−0.02	0.02	0.05	0.06
14. *DRD4* genotype	0.10	0.02	−0.15*	−0.10	−0.10	0.05	0.13	−0.06	−0.10	−0.10	0.16*	0.11
15. *DAT1* genotype	−0.04	−0.02	0.01	−0.01	−0.07	−0.09	−0.00	−0.07	0.00	0.02	0.07	0.01
16. *BDNF* genotype	−0.04	−0.14	−0.06	−0.05	−0.05	−0.01	−0.04	−0.08	−0.04	−0.04	−0.04	0.06
17. *COMT* genotype	0.07	0.11	−0.07	−0.07	−0.05	0.04	0.05	−0.05	−0.05	−0.05	−0.07	−0.05

*p < 0.05, **p < 0.01, ***p < 0.001.

### Three-Way Interaction Models

In the complete-case analysis, the G × E_1_ × E_2_ interaction effect emerged significant (*β* = −17.17, SE = 3.50, *p* < 0.0001). However, this was not replicated in the imputed analysis (*β* = −0.87, SE = 1.31, *p* = 0.51). Although both the complete-case and imputed data analyses had relatively large in-sample effect sizes (*R*^2^ = 0.31 and 0.26, respectively), their out-of-sample effect sizes were very low (LOOCV *R*^2^ = −0.16 and 0.07, respectively), indicating poor generalization. The negative LOOCV *R*^2^ and the fact that the three-way interaction effect was only significant in the non-imputed analysis are strongly suggestive of model overfitting. For these reasons, we reran all analyses without the three-way interaction term but retaining all two-way (i.e., G × E^1^, G × E_2_, E_1_ × E_2_) interaction terms. Results of the three-way interaction models are shown in Table 3.

**Table 3:** Predicting toddler attentional competence at 18 and 24 months based on the three-way interaction of prenatal adversity, dopamine-related genes, and early maternal parenting behaviors with/without imputation.

	**Without Imputation**	**With Imputation**
	Nobs = 134, *N* = 237	Nobs = 197, *N* = 394
**Predictors**
Intercept	−0.05	−0.01
24 months present	0.33*	0.40***
Maternal age	0.04*	0.03*
Postnatal depression	−0.21**	−0.28***
Boys	−0.14	−0.23*
Maternal education (college)	−0.18	−0.30
Maternal education (university)	−0.15	−0.37*
Prenatal adversity (E_1_)	0.60**	0.03
Dopamine-related genes (G)	−1.19**	−0.79**
Early maternal parenting (E_2_)	1.91***	0.45*
E_1_ × G	−1.25	−1.26***
E_1_ × E_2_	3.00***	1.01***
G × E_2_	−7.23***	−5.07***
G × E_1_ × E_2_	−17.17***	0.87
G		
*DRD2*	0.21***	0.00
*DRD4*	0.02	0.05
*DAT1*	0.05	0.11*
*BDNF*	0.57***	0.35***
*COMT*	0.16***	−0.49***
E_1_		
Prenatal depressive symptoms	0.16	0.00
Prenatal stressful life events	0.40***	0.85***
Birth weight (g)	−0.44***	−0.14
E_2_	0.19	0.00
Infant-related attention	0.28***	0.22***
Tactile stimulation	−0.02	0.30***
Vocalization	0.23	0.41***
Infant-related activities	0.28***	0.08*
Maternal sensitivity		
*R* ^2^	0.31	0.26
LOOCV *R*^2^	−0.16	0.07

*p < 0.05, **p < 0.01, ***p < 0.001.

### Two-Way Interaction Models

**Figure 1:**
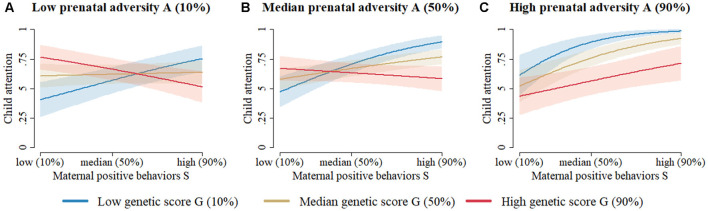
The prediction of toddler attentional competence at 18–24 months based on the two-way interaction model (Table 4) without missing data imputation and without variable selection (column 1). **(A)** When prenatal adversity is low, attentional competence of young children with low dopaminergic genetic scores increases from low to high with increasingly positive early maternal parenting behavior. Meanwhile, children with moderate or high dopaminergic genetic scores start at a relatively high level of attentional competence, which seems to be unaffected by an increase in early positive maternal parenting behaviors. **(B)** When prenatal adversity is moderate, young children with low dopaminergic genetic scores start at moderate levels of attentional competence, which rapidly increases as positive early maternal parenting behavior increases. Meanwhile, children with moderate or high dopaminergic genetic scores start at a high level of attentional competence, which seems to be unaffected by an increase in early positive maternal parenting behaviors. **(C)** When prenatal adversity is high, young children with low dopaminergic genetic scores start at a high level of attentional competence, which seems relatively unaffected by increasingly positive early maternal parenting behaviors. Children with moderate dopaminergic genetic scores initially have moderate levels of attentional competence, which increases linearly as positive early maternal parenting behavior increases. Children with high dopaminergic genetic scores initially have low levels of attentional competence, which also increases linearly as early positive maternal parenting behaviors increase. *Note*. Figures for the two-way interaction models with/without imputation and with/without variable selection all look very similar. *Note 2*. Despite the absence of a three-way interaction effect, results must be graphically represented similarly to a three-way interaction model, since all three main components (i.e., G, E_1_, and E_2_) interact with one another in two-way interactions within the same model.

Results of the two-way interactions models are shown in Table 4. All two-way interaction effects were significant in both the complete-case and imputed analyses (*p* < 0.0001). In the complete-case full model, *DAT1* (*β* = 0.15, SE = 0.05, *p* = 0.002), *BDNF* (*β* = 0.25, SE = 0.12, *p* = 0.04), and *COMT* (*β* = −0.52, SE = 0.09, *p* < 0.0001) seemed to be the most important genetic drivers of the observed interaction effects. Among the prenatal adversity factors, maternal stressful life events emerged as most important for the interaction (*β* = 0.87, SE = 0.15, *p* < 0.0001). Regarding early maternal parenting, tactile stimulation (*β* = 0.22, SE = 0.08, *p* = 0.01), vocalization (*β* = 0.32, SE = 0.09, *p* = 0.0003), and infant-related activities (*β* = 0.40, SE = 0.11, *p* = 0.0003) seemed to be the most relevant in interacting with dopamine-related genes or with prenatal adversity. The model had moderate effect size (in-sample *R*^2^ = 0.32, LOOCV *R*^2^ = 0.03), interaction effects are visualized in Figure 1.

**Table 4:** Predicting toddler attentional competence at 18 and 24 months based on two-way interactions between prenatal adversity, dopamine-related genes, and early maternal parenting behaviors with/without imputation and with/without variable selection.

	Without Imputation		With Imputation	
	Nobs = 134, *N* = 237		Nobs = 197, *N* = 394	
Predictors	All	Best choice	All	Best choice
Intercept	0.19	0.52	−0.10	−0.02
24 months present	0.31*	0.30*	0.40***	0.40***
Maternal age	0.03	0.02	0.04**	0.03*
Postnatal depression	−0.35***	−0.31***	−0.28***	−0.28***
Boys	−0.31*	−0.37**	−0.22*	−0.24*
Maternal education (college)	−0.34	−0.28	−0.29	−0.31
Maternal education (university)	−0.25	−0.25	−0.36*	−0.37*
Prenatal adversity (E_1_)	0.11	0.06	0.06	0.03
Dopamine-related genes (G)	−0.91*	−0.44	−0.85**	−0.83**
Early maternal parenting (E_2_)	0.69*	0.55*	0.51**	0.40*
E_1_ × G	−2.02***	−1.44***	−1.31*	−1.09***
E_1_ × E_2_	1.48***	0.85**	1.00***	0.83***
G × E_2_	−6.34***	−2.99***	−5.67***	−4.88***
G				
*DRD2*	−0.04		0.02	
*DRD4*	0.03		0.06	
*DAT1*	0.15**	0.30**	0.10*	0.12**
*BDNF*	0.25*		0.35***	0.37***
*COMT*	−0.52***	−0.70***	−0.47*	−0.50***
E_1_				
Prenatal depressive symptoms	0.01		−0.00	
Prenatal stressful life events	0.87***	1***	0.84***	1***
Birth weight (g)	−0.12		−0.16	
E_2_				
Infant-related attention	−0.02		−0.00	
Tactile stimulation	0.22*		0.24***	0.22***
Vocalization	0.32***	0.40***	0.30***	0.30***
Infant-related activities	0.40*	0.50**	0.40***	0.41***
Maternal sensitivity	0.04	0.09	0.07*	0.07*
				
*R* ^2^	0.32	0.41	0.25	0.25
LOOCV *R*^2^	0.03	0.17	0.10	0.14

*p < 0.05, **p < 0.01, ***p < 0.001.

In the variable selection model, *DAT*1 (*β* = 0.30, SE = 0.07, *p* < 0.0001), and *COMT* (*β* = −0.70, SE = 0.11, *p* < 0.0001) were retained for the genetic component; prenatal stressful life events (*β* = 1) for the adversity component; and maternal vocalization (*β* = 0.40, SE = 0.12, *p* = 0.0008), maternal infant-related activities (*β* = 0.50, SE = 0.15, *p* = 0.001), and maternal sensitivity (*β* = 0.09, SE = 0.05, *p* = 0.10) for the early maternal parenting component. The effect size of the model with variable selection was moderate (in-sample *R*^2^ = 0.41 and LOOCV *R*^2^ = 0.17).

A very similar picture emerged in the imputed models. In the full model without variable selection, maternal sensitivity emerged as an additional important early parenting behavior for the observed interactions (*β* = 0.07, SE = 0.03, *p* = 0.03). In the variable selection model, *DAT1* (*β* = 0.12, SE = 0.04 *p* = 0.005), *BNDF* (*β* = 0.37, SE = 0.10, *p* = 0.0002) and *COMT* (*β* = 0.50, SE = 0.07, *p* < 0.0001) were retained for the genetic component; maternal stressful life events for the adversity component (*β* = 1); and maternal sensitivity (*β* = 0.07, SE = 0.03, *p* = 0.03), tactile stimulation (*β* = 0.22, SE = 0.06, *p* = 0.0006), vocalization (*β* = 0.30, SE = 0.07, *p* < 0.0001), and infant-related activities (*β* = 0.41, SE = 0.10, *p* < 0.0001) for early maternal parenting. Effect sizes of both models were moderate (in-sample *R*^2^ = 0.25, LOOCV *R*^2^ = 0.10 for the full model; in-sample *R*^2^ = 0.25, LOOCV *R*^2^ = 0.14 for the model with variable selection).

## Discussion

In a prospectively followed prenatal cohort, we examined the complex interplay between three important forces of attention development: 1) genetic variations in the dopaminergic pathway (using a genetic score composed of five dopamine-related genes), prenatal adversity (captured through children’s birth weight, the presence of prenatal maternal depressive symptoms and stressful life events), and the earliest rearing environment (captured through a range of observed maternal parenting behaviors). Our study benefitted from a sample with rich measures on prenatal adversity, dopamine-related gene variants, observational measures of maternal parenting behavior, and repeated assessments of toddlers’ attentional competence. A further strength of our study was the use of a statistical approach (LEGIT) to simultaneously analyze complex G × E interactions, which provides greatly enhanced power over traditional models that analyze a single G × E effect at a time.

Our hypothesis of finding a three-way interaction effect for prenatal adversity, dopamine-related genes, and early maternal behavior on toddlers’ attentional competency was not confirmed. Although the complete case analysis indicated the presence of such an interaction effect, the model did not generalize, and when imputing missing observations, the interaction effect was not significant anymore. These observations point to possible model overfitting, especially in smaller samples. In line with this, when we reran the analysis without the three-way interaction term, a more consistent picture emerged. Significant two-way interaction effects emerged for prenatal adversity by dopamine-related genes; prenatal adversity by early maternal behavior; and dopamine-related genes by early maternal behavior on toddler attentional competence in both the complete case analysis and analysis with imputation for missing data. Furthermore, the in- and out-of-sample effect sizes also indicated that the model generalizes.

Our findings suggest that multiple dopamine-related genes interact with prenatal adversity to predict toddler attentional competence. Based on our models, *DAT1*, *COMT*, and *BDNF* emerged as the most significant among the genes tested. Previously, *DAT1* has been one of the most consistently implicated candidate genes in relation to ADHD by linkage, association, and meta-analytic studies (Sharp et al., 2009). Importantly, *DAT1* genotype has been linked to variation in both cognitive and neurobiological measures of attention (Gizer et al., 2009; Sharp et al., 2009). Moreover, a number of environmental factors have been hypothesized to moderate the effect of *DAT1* on ADHD-related phenotypes (Franke and Buitelaar, 2018). Some of these include prenatal factors, such as maternal smoking and alcohol use during pregnancy, prenatal maternal stress, and birth weight (for a review, see Franke and Buitelaar, 2018). Contrary to our findings, the single study that looked at *DAT1* by prenatal stress interaction effect on ADHD reported a lack of such effect (Grizenko et al., 2012). However, that study used a retrospective design to collect information on prenatal maternal stress when children were 6–12 years old. The two studies that assessed interactions of *DAT1* with birth weight reported nominally significant effects on the occurrence of conduct problems in children with ADHD in a case-only study (Langley et al., 2008) and significant G × E effects for a genetic index including *DAT1, DRD4*, and *DRD2* and birth weight on ADHD symptoms in a sibling sample (Jackson and Beaver, 2015). Importantly, certain aspects of parenting were also shown to interact with *DAT1* in relation to ADHD-related phenotypes. These include parental expressed emotions, negative and positive parenting practices, and maternal warmth (for a review, see Franke and Buitelaar, 2018). In summary, our study supports prior evidence for the involvement of *DAT1* in ADHD-related phenotypes in interaction with either the prenatal or postnatal environment.

Although a recent meta-analysis did not confirm the main effect of *COMT* gene variants on ADHD, it could not rule out the importance of *COMT* in combination with other factors (Sun et al., 2014). Indeed, in a combined analysis of two large cohorts (ALSPAC and PREDO), prenatal anxiety and child *COMT* genotype predicted ADHD symptoms at multiple time points (O’Donnell et al., 2017). In addition, *COMT* genotype also seemed to interact with prenatal maternal smoking to predict aggressive behavior and autistic symptoms in children with ADHD (Nijmeijer et al., 2010; Brennan et al., 2011) and interact with birth weight to predict antisocial behavior in children with ADHD (Thapar et al., 2005). Interactions of the *COMT* gene with parenting behavior have not been investigated to our knowledge in relation to ADHD. In summary, our findings are in line with previous literature suggesting an interplay between COMT and the prenatal environment to shape ADHD-related phenotypes. Furthermore, we add to the existing literature by showing that a genetic index including *COMT* interacts with maternal parenting behavior to affect the attentional competence of young children.

Variants in the *BDNF* gene have also been implicated in ADHD-related phenotypes both as exerting a main effect (Langley et al., 2008; Li et al., 2014; Luo et al., 2020) and in interaction with environmental stressors, such as early deprivation or family SES (Lasky-Su et al., 2007; Gunnar et al., 2012) in both European and Asian populations. G × E studies of ADHD-related phenotypes involving the *BDNF* gene, however, are still rare. One interesting study examined the interaction of the *BDNF* Val66Met polymorphism and parenting in children (aged 6–15 years) diagnosed with ADHD and found a significant interaction effect for child *BDNF* by mothers’ positive feelings about caring in relation to the development of internalizing comorbidities (Park et al., 2014).

We further found that prenatal adversity interacted with both dopamine-related genes and maternal parenting behavior in affecting toddler attentional functioning. There is growing evidence for the involvement of prenatal adversity in the risk for developing ADHD-related phenotypes (Glover, 2011; Graignic-Philippe et al., 2014), although there is currently insufficient support for a causal relationship (Sciberras et al., 2017). The most commonly researched adversities in relation to ADHD include maternal prenatal smoking, alcohol and substance use, maternal stress, and offspring birth weight (Morgan et al., 2018). Unfortunately, we were unable to investigate the effects of prenatal smoking, alcohol, and substance use as these variables had an extremely large proportion of missing data in our cohort. Nevertheless, we did examine the effects of prenatal maternal stress and birth weight, as well as maternal prenatal depressive symptoms, which have also been consistently implicated in the development of maladaptive child outcomes (Madigan et al., 2018). Of all prenatal adversities considered here, maternal prenatal stress seemed to be the most relevant component when considering offspring dopamine-related genes. This finding is partly in line with a recent study that reported significant G × E effects for prenatal maternal stress and children’s *DRD4* genotype but not *DAT1* on ADHD symptoms (Grizenko et al., 2012). However, as both this and our study show, not all children exposed to prenatal adversity will experience later difficulties. Constitutional characteristics, such as genetic variation may be key in determining who will be more susceptible to the deleterious effects of the environment, as is contended by the diathesis-stress or differential susceptibility hypotheses (Belsky, 1997, 2005; Ingram and Luxton, 2005).

Another noteworthy finding of this study is that early positive maternal behavior seemed to buffer the effect of both prenatal adversity and genetic susceptibility although not their joint effect on toddler attentional competence. The observation that positive maternal behavior may attenuate both environmental and genetic risks is in line with previous literature (Sonuga-Barke and Harold, 2018) and has important consequences for guiding interventions such as behavioral parent training programs for families with ADHD. Despite the strong evidence in support of a biological basis for ADHD symptoms, researchers have speculated that the child’s environment may play a particularly salient role in determining outcomes for children with ADHD, even if environmental factors may not be the primary cause of their core symptoms (Barkley, 2006). During infancy, the caregiver provides much of the child’s attention regulation through orienting (Posner et al., 2014). This external control eventually becomes internalized as toddlers gradually gain control over their own emotional and cognitive states through self-regulation (Posner et al., 2014). Therefore, understanding the ways in which parents can help their children better regulate their attention, emotions, and behavior is going to be invaluable for the success of behavioral parent training programs, for parents typically play a major role in changing their child’s behavioral symptoms (e.g., through parent training and behavior therapy programs; Johnston and Mash, 2001; Deault, 2010). A newly emerging field of “therapy genetics” has produced some promising results to this end. In one study among a large group of toddlers with externalizing problems, the largest effect for a video-feedback-based intervention promoting positive parenting and sensitive discipline was found in children carrying the *DRD4* 7R allele (Bakermans-Kranenburg et al., 2008). In another smaller pilot study of children with ADHD, the largest effects of a behavioral parent training program were seen in children not homozygous for the *DAT1* 10R allele (van den Hoofdakker et al., 2012).

Our study also pinpointed a number of specific maternal behaviors that were linked with improved attentional competence in toddlers, such as maternal sensitivity, tactile stimulation, vocalization, and activities including play and grooming. These behaviors emerged from coded observations of mother-child interactions rather than maternal self-reports. The over-reliance on self-report questionnaires for assessing parenting behavior may limit both the validity and reliability of the parenting behaviors being assessed. In addition, most prior studies tended to isolate one or two parenting behaviors, rather than examining several parenting measures simultaneously to explore if more robust associations exist that go beyond specific measures of parenting (Deault, 2010).

ADHD has classically been viewed as a primarily fixed cognitive “deficit,” mainly underlined by genetic and neurobiological mechanisms (Barkley, 1990; Weiss and Hechtman, 1993; Hinshaw, 1994). However, this view falls short in accounting for the way environmental and biological risk factors seem to interact to produce the diverse developmental pathways, clinical outcomes, and frequent comorbidities observed in ADHD (Mannuzza et al., 2004; Castellanos et al., 2005; Halperin et al., 2008). As a result, researchers have recently turned to the biopsychosocial framework to better explain the complex developmental processes underlying the pathophysiology of ADHD (Singh, 2008). Contrary to the fixed deficit model, the biopsychosocial theory posits that ADHD is caused by the interplay of genetic and environmental influences that occur throughout development in underlying neurobiological systems (Sonuga-Barke, 1998, 2009). Accordingly, the original risk for developing the disorder can be moderated by later factors that alter the trajectory of development for better or worse (Taylor, 1999; Singh, 2008). Understanding these moderating influences—both protective and harmful—is essential for predicting key features of the disorder, such as its emergence, persistence, offset, and the frequent development of comorbidities (Sonuga-Barke, 2009). In line with this thinking, here we reported that certain positive aspects of the early maternal behavior moderated the negative impact of both prenatal adversity and genetic susceptibility on toddlers’ attentional competence, albeit not their joint effect.

Although our findings were mainly interpreted in relation to the pathophysiology of ADHD, it was done so, since the overwhelming majority of available G × E studies that examined interactions between the very environmental exposures and genetic variants we considered in this study, focused on ADHD-related deficits in attention. However, it is important to note that attention deficits are present in numerous other psychiatric disorders, such as schizophrenia, bipolar disorder, mood disorders, and autism spectrum disorder to name a few (Burack et al., 2017). Furthermore, dysfunctions in the dopamine system that are related to the gene variants we considered here are also implicated, amongst others, in schizophrenia, bipolar disorder, Parkinson’s disease, phenylketonuria, and autism spectrum disorder (Diamond et al., 1997; DiCarlo et al., 2019; Nieoullon, 2002; Hayden and Nurnberger Jr, 2006; Scheggia et al., 2012; Mandolini et al., 2019; Pigoni et al., 2019). We plan on following our participants to see if lower attentional competence early in life will evolve into cognitive and psychopathological problems later on.

Inevitably, we were faced with a number of limitations. First, obtaining rich measures and detailed coding of maternal behavior meant that we had to compromise regarding the sample size. However, as Jolicoeur-Martineau et al. (2019) previously demonstrated, LEGIT performs well with sample sizes similar to that of the current study. Second, we assessed maternal prenatal depressive symptoms at a single time point. Consequently, this prevented us from examining the effect of timing and chronicity, the latter of which is a known modifier of the effect of maternal depressive symptoms on child outcomes (Brennan et al., 2000; Hammen and Brennan, 2003; Lahti et al., 2017; Tuovinen et al., 2018). Third, toddlers’ attentional competency was rated by the mothers. This can be problematic when mothers are also reporting on their own mood symptoms. Nevertheless, our study benefitted from using observational measures of early maternal parenting behaviors, which were rated by trained coders blind to the mothers’ prenatal depressive symptoms and offspring attentional competency. Furthermore, the ITSEA used to assess toddlers’ attentional competency is a valid parent-report measure (Carter et al., 2003), which is less prone to measurement error. Parents are asked to report on what is present, i.e., their child’s everyday activities that are indicative of the level of their attentional functioning (e.g., *“Plays with toys for 5 min or more.”*), rather than what is absent, i.e., deficits in their children’s attentional functioning. The assessment of ADHD can be challenging in the early years, thus recognition of important developmental processes, such as attentional competence can be a useful guide to the types of processes that are likely precursors to the disorder (Deault, 2010). As our young participants become older and increasingly capable of understanding verbal task instructions, we aim to repeat these analyses using laboratory-based assessments of child attention.

### Implications

As, we have seen here, prenatal adversity can render genetically susceptible children to exhibit lower attentional competence already in toddlerhood, while a positive early rearing environment facilitates the development of children’s attentional competence. Therefore, standard prenatal care should include components that target women’s psychological well-being during pregnancy. At the same time, interventions for children with a high susceptibility for developing attentional problems might benefit from promoting positive parenting practices. In addition, these findings underscore the importance of including measurement of the psychosocial environment of the child in line with the biopsychosocial formulation of mental disorders, even when studying neurodevelopmental disorders or related processes (White et al., in press). Furthermore, future research should combine longitudinal developmental cohorts with similar available measures to investigate the complex interplay between the various genetic and environmental components that act to produce complex phenotypes. The computational tools necessary to investigate such complex interactions are now readily available to researchers.

## Data Availability Statement

The data for this study will be shared upon request to the corresponding author.

## Ethics Statement

The studies involving human participants were reviewed and approved by REB, Douglas Institute of Health, Montreal, Canada. Written informed consent to participate in this study was provided by the participants’ legal guardian/next of kin.

## Author Contributions

LA, RL, MS, JL, AF, and JK were responsible for study design, data collection, and revision of manuscript. ES, AJ-M, and AW were responsible for study design, analysis, drafting, and revision of manuscript. All authors contributed to the article and approved the submitted version.

## Conflict of Interest

The authors declare that the research was conducted in the absence of any commercial or financial relationships that could be construed as a potential conflict of interest.

## Publisher’s Note

All claims expressed in this article are solely those of the authors and do not necessarily represent those of their affiliated organizations, or those of the publisher, the editors and the reviewers. Any product that may be evaluated in this article, or claim that may be made by its manufacturer, is not guaranteed or endorsed by the publisher.
