# Supplementary material for: The Interplay Between Prenatal Adversity, Offspring Dopaminergic Genes, and Early Parenting on Toddler Attentional Function
Source: Front Behav Neurosci. 2021 Jul 29;15:701971. doi: 10.3389/fnbeh.2021.701971 (PMC8370126; doi:10.3389/fnbeh.2021.701971)

**Supplemental Material**

**The interplay between prenatal adversity, offspring dopaminergic genes, and early parenting on toddler attentional function**

**Additional information concerning the calculation of G, E_1_, E_2_ and their interactions using LEGIT.**

The actual models used in Table 3 can be written in the following way:

$y= \beta_{0}+\beta_{1}c_{1}+\beta_{2}c_{2}+\beta_{3}c_{3}+\beta_{4}c_{4}+\beta_{5}c_{5}+\beta_{6}c_{6}+\beta_{E1}E_{1}{+ \beta}_{G}G+\beta_{E2}E_{2}+\beta_{E1xG}E_{1}G+\beta_{E1xE2}E_{1}E_{2}+\beta_{GxE2}{GE}_{2}$,

where $y$ is the attention outcome and $c_{1}, c_{2}, c_{3},c_{4}, c_{5}, c_{6}$ are the covariates: 24 months, maternal age, postnatal depression, boys, college education, and university education. The $\beta_{i}$ coefficients are estimated by LEGIT and correspond to the values reported in the Table.

For $G$, we have that:

$$G= p_{1}DRD2+p_{2}DRD4+p_{3}DAT1+p_{4}BDNF+p_{5}COMT.$$

The coefficients $p_{1}, p_{2}, p_{3},p_{4}, p_{5}$ are estimated by LEGIT and and corresponds to the values reported in the Table. Note that the model is constraint so that ${|p}_{1}|+ \left| p_{2} \right|+ \left| p_{3} \right|+\left| p_{4} \right|+ \left| p_{5} \right|=1$, which means that the parameters are the relative contributions of each genetic variable.

For $E_{1}$, we have that:

$$E_{1}= q_{1}depression+q_{2}stressful\_life\_events+q_{3}birthweight.$$

The coefficients $q_{1}, q_{2}, q_{3}$ are estimated by LEGIT and and corresponds to the values reported in the Table. Note that the model is constraint so that ${|q}_{1}|+ \left| q_{2} \right|+ \left| q_{3} \right|=1$, which means that the parameters are the relative contributions of each environmental variable.

For $E_{2}$, we have that:

$$E_{2}= r_{1}attention+r_{2}tactile+r_{3}vocal+r_{4}activities+r_{5}maternal\_sensitivity.$$

The coefficients $r_{1}, r_{2}, r_{3},r_{4}, r_{5}$ are estimated by LEGIT and and corresponds to the values reported in the Table. Note that the model is constraint so that ${|r}_{1}|+ \left| r_{2} \right|+ \left| r_{3} \right|+\left| r_{4} \right|+ \left| r_{5} \right|=1$, which means that the parameters are the relative contributions of each environmental variable.

LEGIT is able to estimate all parameters by alternating between 1) predicting the $\beta_{i}$ parameters, while leaving the other parameters constant, 2) predicting the $p_{i}$ parameters, while leaving the other parameters constant, 3) predicting the $q_{i}$ parameters, while leaving the other parameters constant, and 4) predicting the $r_{i}$ parameters, while leaving the other parameters constant.

A simpler example with only a latent G (instead of latent G, E_1_, and E_2_) can be found below showing how alternating optimization works:

**Table 5:** *Example of alternating optimization being used to estimate a simple G×E model with 4 genetic variables and 1 environment variable; E*[***y*]** *= β_0_ + β_g_* ***g*** *+ β_e_ e* + *β_eg_* *e****g*** *with a single e and* ***g*** *= p_1_g_1_* + *p_2_g_2_* + *p_3_ g_3_* + *p_4_g_4_*.

| *Parameters*  *Step* | *β_0_* | *β_g_* | *β_e_* | *β_eg_* | *p_1_* | *p_2_* | *p_3_* | *p_4_* | *R^2^* |
| --- | --- | --- | --- | --- | --- | --- | --- | --- | --- |
| 0 - initialization | ? | ? | ? | ? | **.25** | **.25** | **.25** | **.25** | ? |
| 1 - main | **5.12** | **-.77** | **3.05** | **-.90** | .25 | .25 | .25 | .25 | .843 |
| 1 - genes | 5.12 | -.77 | 3.05 | -.90 | **.87** | **-.47** | **1.56** | **-.93** | .947 |
| $\sum_{j=1}^{k} \left\vert p_{j}\right\vert=1$ | 5.12 | **-2.96** | 3.05 | **-3.46** | **.23** | **-.12** | **.41** | **-.24** | .947 |
| 2 - main | **4.99** | **-1.83** | **3.05** | **-3.84** | .23 | -.12 | .41 | -.24 | .952 |
| 2 - genes | 4.99 | -1.83 | 3.05 | -3.84 | **.23** | **-.13** | **.40** | **-.25** | .952 |
| $\sum_{j=1}^{k} \left\vert p_{j}\right\vert=1$ | 4.99 | **-1.86** | 3.05 | **-3.90** | **.22** | **-.13** | **.40** | **-.25** | .952 |
| **…** | **…** | **…** | **…** | **…** | **…** | **…** | **…** | **…** | **…** |
| End | 4.95 | -1.87 | 2.96 | -3.91 | .21 | -.14 | .39 | -.26 | .953 |
| Optimal | 5 | -2 | 3 | -4 | .20 | -.15 | .40 | -.25 |  |

**Figure S1.** Schematic representation of the parameters and their relations as included in the three-way interaction model


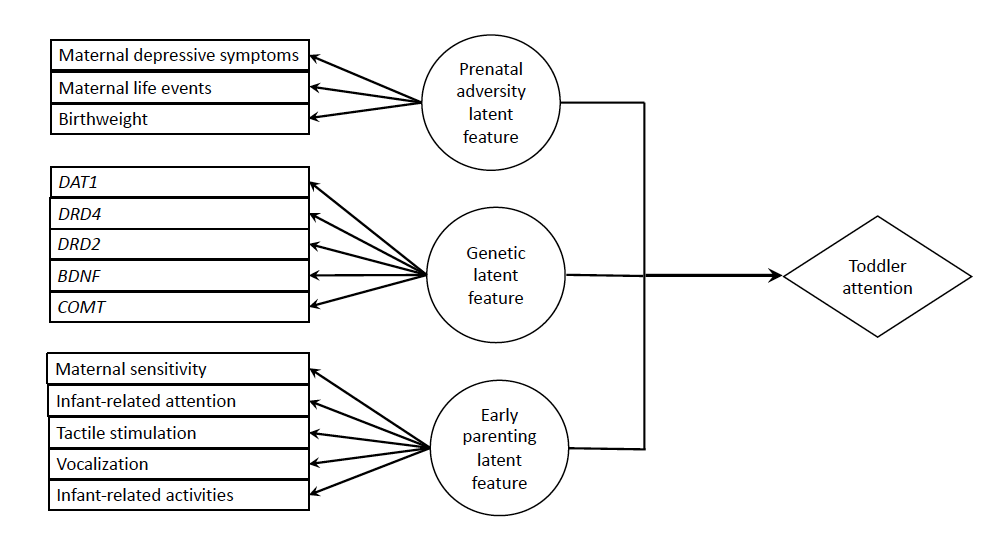

Supplement: Supplementary file 1 [file Data_Sheet_1.docx]
